# Multiple cystic/cavitated metastases

**DOI:** 10.36416/1806-3756/e20240173

**Published:** 2024-08-07

**Authors:** Edson Marchiori, Bruno Hochhegger, Gláucia Zanetti

**Affiliations:** 1. Universidade Federal do Rio de Janeiro, Rio de Janeiro (RJ) Brasil.; 2. University of Florida, Gainesville (FL) USA.

**Figure 1:**
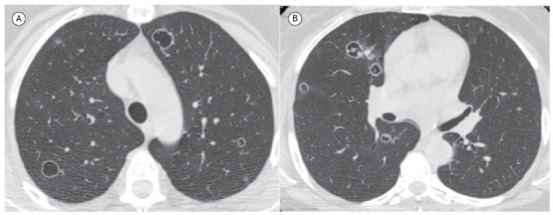
CT scans with lung window settings at the level of the upper lobes (in A) and lung bases (in B) showing multiple nodules of varying sizes, many of them cavitated, predominantly with peripheral distribution. Note solid content inside some of the nodules.

A 58-year-old female patient diagnosed with cervical carcinoma reported dyspnea and cough for about one month. A CT of the chest (Figure 1) showed multiple cystic/cavitated nodular images in both lungs.

Cavitated lesions can be the result of a series of pathological processes, especially necrosis, and is defined as a gas-filled space, evidenced on CT as an area of low attenuation within a lung consolidation, mass, or nodule. The differential diagnosis of multiple cavitated nodular lung lesions is broad, including infectious diseases such as septic embolism, tuberculosis, and fungal and parasitic infections, as well as neoplastic lesions (metastases, lymphoma, etc.), in addition to other less common etiologies (tracheobronchial papillomatosis, rheumatoid nodules, Wegener’s granulomatosis, and nodular amyloidosis, among others). The most common causes are septic embolism and cavitated metastases.

Septic embolism occurs due to embolization of fragments infected with microorganisms into the lungs. The disease is most commonly secondary to right endocarditis or septic thrombophlebitis, but it may occur secondary to infected endovascular catheters, suppurative processes of the skin, head, or neck, or contamination related to the use of intravenous drugs. CT imaging reveals multiple bilateral nodules, well or poorly defined, predominantly with peripheral distribution, showing varying degrees of cavitation. Associated peripheral triangular images often correspond to infarcts due to vascular occlusion. Septic embolism can occur together with unilateral or bilateral pleural effusion.^1^ ^-^ ^3^

Cavitated metastases occur most commonly in squamous cell carcinomas, corresponding to 70% of the cases on average. Head and neck tumors, pelvic tumors (uterus, ovary, prostate), and adenocarcinomas of the large intestine are the most common primary sites, although any primitive tumor, in principle, can give rise to cavitated metastases. In metastases, the cavitations originate both from tumor necrosis and from the formation of a valvular mechanism due to neoplastic infiltration into the distal airways. The walls of the cavitations are most often thick and irregular, but they can also be thin, similar to cysts. Clinical aspects are very important for the differential diagnosis. Septic embolism clinically presents with fever, dyspnea, cough, and pleuritic pain. Blood culture may be positive. The presence of a previously known primary tumor may lead to the suspicion of pulmonary metastases. Patients with metastases are often asymptomatic from a respiratory point of view. Our patient had a previous diagnosis of cervical carcinoma. The final diagnosis was cavitated metastases from uterine carcinoma.^1^ ^-^ ^3^
